# Ethics Guideline Development for Neuroscience Research involving Patients with Mental Illness in Japan

**DOI:** 10.1007/s41649-023-00240-x

**Published:** 2023-02-10

**Authors:** Yoshiyuki Takimoto, Akifumi Shimanouchi

**Affiliations:** 1 Department of Biomedical Ethics, The University of Tokyo, Tokyo, Japan; 2 Department of Philosophy, Hoshi University, Tokyo, Japan

**Keywords:** Mental illness, Informed consent, Neuroethics, Research ethics, Relational autonomy, Dual burden, Minimal risk

## Abstract

This study aims to develop guidelines of key concepts and specific considerations to make the research more ethical when conducting neurological examinations and treatment interventions in mentally ill patients. We analyzed guideline development theory and literature, previous issues, and discussions with specialists of philosophy, medicine, sociology, and bioethics. The selection of research participants, drafting of intervention plans, and informed consent process were examined with reference to the dual burden; the minimal risk as a general rule of ethical allowance levels, assent and dissent to assess the individual’s judgment capacity for consent, relational autonomy for personal consent with assistance by the proxy, and risk/benefit assessments. When conducting studies, this guideline requires that these three processes be set up appropriately on a case-by-case basis.

**Supplementary Information:**

The online version contains supplementary material available at 10.1007/s41649-023-00240-x.

## Introduction

A careful and prudent approach is necessary when medical research involves patients with mental illness. This is due to the significant issue of vulnerability, a concept touched on in the Helsinki Declaration (1964, last revised in 2000). Increased ethical considerations are necessary for these research participants. However, to the best of our knowledge, there are no detailed universal guidelines that clearly state the ethical considerations and research ethics required for medical research that includes patients with mental or neurological disorders as research participants. This research is an unavoidable reality in the field of neuroscience.

For research into psychiatric and neurological disorders, respect for persons is the most important of the three principles of research ethics (i.e., respect for persons, beneficence or non-maleficence, and justice). This point is relevant because it is conceivable that informed consent might be difficult to obtain appropriately from a research participant if the individual’s decision-making capacity is impaired. Characteristics that require special consideration for research participation, such as psychiatric and neurological disorders, are known as “vulnerabilities.” These vulnerabilities create a sharper ethical dilemma between the need to protect research participants and the societal benefits of participation, such as future benefits of the research to patients with the same disease, as is typical for general medical research that requires special consideration for study participants by researchers (Rosenstein and Miller 2008).

In brain science research, it is inevitable that research is conducted on mentally ill patients with such “vulnerabilities.” The guidelines were developed as part of a research project supported by the Strategic Research Program for Brain Sciences (SRPBS) of the Japan Agency for Medical Research and Development (AMED), the funding agency for Japanese medical research—analogous to the NIH in the United States. The SRPBS by AMED involves leading researchers, research centers, and research institutions in Japan related to brain science research. Unlike the United States, Japan has no laws that comprehensively regulate medical research, with the exception of research in specific fields, such as regenerative medicine and clinical trials, and research ethics is addressed by adhering to guidelines. Failure to comply with the guidelines results in financial penalties from the AMED, which manages public research funds. These guidelines were developed by researchers in philosophy, social sciences, and medicine who are capable of dealing with bio/medical ethics, including research ethics, under the direction and supervision of the principal investigator of the Ethical, Legal and Social Implications (ELSI) Research Group of the SRPBS. Its purpose is to serve as a practical research guideline that can be used in the field of brain science research by researchers, research centers, and research institutions participating in the SRPBS, not only to protect research participants, but to promote appropriate brain science research.

## Methods

The authors have been working on the study of theoretical constructs concerning guideline development for individuals with mental illness for 4 years. As part of the preparations for these guidelines, in the early stages of ELSI research at the SRPBS, we conducted preliminary theoretical research (review of basic literature on neuroethics in the Anglo-America region and consolidation of issues) (Dukoff and Sunderland 1997; Goodwin 2016; Levin et al. 2004; Binik 2014), ensured the establishment and operation of a research ethics consultation service (uncovering ethical issues related to brain science research), and facilitated science communication between citizens and experts on brain science research (surveying the awareness of both experts and citizens on brain science research). These steps helped lay the foundation for guideline development. All individuals engaged in the preparation and writing of the guidelines are researchers capable of dealing with bio/medical ethics, including research ethics, and have diverse academic backgrounds. The four members met once a month for 2 years to discuss the format of the guidelines and the selection of topics to be covered in the initial stages. Then, each of them presented the contents of the topics they were in charge of and they all discussed and examined the contents, reflected the discussion in the written contents before the next meeting, until the final draft of the guidelines was completed. Based on the final draft, revisions were made through an exchange of opinions with two SRPBS program officers and two brain science researchers.

All subjects in this research provided their informed consent. All procedures followed were in accordance with the ethical standards of the Helsinki Declaration (1964, last revised in 2000) of the World Medical Association.

## Results

The ethics guidelines consist of two sections and five chapters, as demonstrated in Table 1. Of the two sections, subsection (B) summarizes the current state of the Japanese ethics review boards and research for ethics support, clinical research laws, etc. This paper summarizes the first section of the guidelines, with an emphasis on (a) the selection of research participants, (b) the drafting of intervention plans, and (c) the informed consent process.

**Table 1:** Guidelines: Table of contents

A. Guidelines for improved research plans
1. Study participant selection
2. Risk/benefit assessments
2–1. Minimal risk
2–2. Minimization of risk
2–3. Maximization of benefits
3. Informed consent
3–1. Background
3–2. Measurement of capacity to provide consent
3–3. Consent from the individual in question
3–4. Assent and dissent
3–5. Consent from proxy
3–6. Privacy protection
4. Special considerations in research participation
4–1. Considerations for research participants
4–2. Consideration for family, acquaintances, and others
B. Guidelines for appropriate ethics review systems
5. Appropriate ethics review
5–1. Ideal ethics review boards
5–2. Research ethics support for researchers
5–3. Compliance with clinical research laws

### Study Participant Selection: Dual Burden

The concept of the dual burden is crucial when selecting patients with mental illnesses as study participants.

#### Dual Burden

In selecting study participants, extra care should be taken because research that is appropriate for non-vulnerable individuals should not be conducted on those with vulnerabilities. This idea was based on the ethical view that those who are already burdened by psychiatric or neurological disorders should not incur an additional burden (the “dual burden”) of research participation (Helmchen 2012). This is derived from the fact that patients with mental and neurological disorders have historically been unethically forced to participate in research (Rothman 1991).

Nevertheless, the ethics guidelines do not prohibit study participation by individuals with psychiatric or neurological disorders. For example, it may be permissible to target vulnerable patients in cases where (1) knowledge about the disease cannot be obtained unless the patient is targeted, (2) the patient with the disease has some capacity to consent and actually wants to participate in the research from the perspective of contributing to society, or (3) the will to participate in research that is deemed to be in the public’s interest is indicated by advance directives of the patient with disease when they were competent. Patients’ participating in research in the public’s interest might be based on Japanese collectivism, rather than for their own medical benefit. Therefore, it is important to avoid creating a double burden through profit inducement. In such cases, risk/benefit assessments and informed consent procedures should be handled with extra care than with cases concerning typical research, with the aim of protecting vulnerable research participants and facilitating their participation.

### Drafting Intervention Plans: Minimal Risk and Risk/Benefit Assessments

If a research plan is scientifically valid and its societal benefits can be anticipated, it is desirable that the suitability of each participant for their involvement in the study be determined with this in mind, considering the personal risks and benefits to each of them (Chwang 2014).

#### Minimal Risk

Risk is divided into three categories: minimal, slightly over minimal, and significantly over minimal (Taylor et al. 2015; Yanos et al. 2009). A crucial point to consider is that a particular medical intervention might not correspond perfectly to any of the above-mentioned categories. In studies involving patients with psychiatric and neurological disorders, the magnitude of risk varies depending on the unique circumstances of the study participants. For example, the invasive intervention of blood sampling is usually considered a minimal risk category. However, if this is performed when an individual with mental illness is in a state of agitation, the patient’s condition might be exacerbated, and the minimal risk category is surpassed.

Research is generally and ethically required to be at the level of minimal risk, but this does not mean that all research that exceeds the minimal risk level is ethically unacceptable. This can be explained using the analogy of risk burdens associated with altruistic acts in daily life. When we attempt to rescue someone who is drowning in the ocean, or who has fallen off a platform at a train station, for example, we take on risks that clearly exceed the minimum level. When lifeguards and train station staff perform similar acts, it is a form of risk burden associated with their work duty. However, when ordinary citizens perform altruistic acts, they take on these risks voluntarily. The act of participating in a study is distinct from treatment; it represents a situation where benefits cannot always be anticipated. Participation in research amid such a situation is an act of altruism that is based on a desire to contribute to the acquisition of knowledge, which can be widely used to realize a societal benefit. The fact that a risk burden that exceeds the minimal risk level is allowed in research participation in the first place is based on the analogy of risk associated with altruistic acts. Nevertheless, as the risk burden must be voluntary, appropriate procedures are required to obtain consent in accordance with the individual’s capacity to understand the situation, even in cases of research on individuals with psychiatric and neurological disorders.

Moreover, even when research that exceeds the minimal risk level must be conducted, efforts to minimize risk are indispensable. In studies of individuals with vulnerabilities, such as patients with psychiatric and neurological disorders, special measures (i.e., additional considerations) should also be taken to minimize risk from the perspective of the individual nature of the risk (dual burden). For example, depending on the circumstances of the study participants, verbal communication of sensations involving itchiness or pain that are associated with interventions, such as when using measurement devices that are affixed to the body, might be impossible. In such instances, special measures are required to ensure that a participant has the “freedom to withdraw” from participation, as for example, through the utilization of buttons that would allow the individual to convey the sensations of itchiness or pain (DuVal  2004; Nugent et al. 2017).

The conditions for approval of a research plan that exceeds the specific minimal risk are: there being no alternative to achieve the objectives of the study than for the person with psychiatric or neurological disorders to participate in the research the high success probability of the research plan, and the societal benefits associated with the significant achievement of the research plan. In other words, the research plan must be judged as balanced, including comparative weighing, in terms of risks and benefits.

#### Risk/Benefit Assessments

In the guidelines, benefits are weighed against the risks and are classified into three categories (2–3): (1) benefits for study participants—(1a) direct benefits and (1b) indirect benefits—and (2) benefits for others (societal benefits) (National Bioethics Advisory Commission 2002). Direct and indirect benefits are considered only for the study participants themselves. In terms of direct benefits (1a), it is necessary to pay attention to therapeutic misconception, in which subjects confuse research with treatment, and seek for the likely benefits to be explained at the time of obtaining informed consent, without eliciting unrealistic expectations. As part of indirect benefits (1b), a sense of well-being that accrues to research participants, based on satisfaction associated with the societal contribution from participation in research, is not limited to patients with psychiatric or neurological disorders. In research targeting vulnerable individuals, such as patients with psychiatric or neurological disorders, as a result of the direct benefits to the research participants, the burden on family members and others who are involved in the care of the vulnerable may be reduced, and this may bring joy to the research participants. Regarding benefits for others (2), the acquisition of accumulated findings could result in the elucidation, prevention, and development of treatment methodologies for mental illnesses. However, this benefit is only a probability, with more detailed risk/benefit assessments required for determining the acceptable level of risk for specific participants in such research, compared to typical clinical studies.

### Informed Consent

Notably, these guidelines emphasize the process of informed assent and dissent within the process of obtaining informed consent for research that involves individuals with vulnerabilities. This is important because the concepts of autonomy and self-determination, as used in typical clinical research, are insufficient. Moreover, the guidelines maintain the concept of “relational autonomy” in the theoretical background (3–1).

#### Assent and Dissent

In the guidelines, the measurement of the capacity to provide consent is categorized as: “understanding,” “appreciation,” “rationality,” and “communicating a choice,” in accordance with one of the standard assessment methodologies proposed by Appelbaum and Grisso (Appelbaum and Grisso 1988). This capacity for consent is used in clinical practice and is assessed mentally and psychologically. Furthermore, the judgment capacity required for study participation is set at a level higher than the capacity needed for medical care, given that benefits do not always accrue to the research participant. This differs from typical medical care scenarios (Appelbaum et al. 1982).

To the extent that research participants have the capacity to provide consent, their consent is required. Since informed consent is a continuous process, it does not end once participant consent is obtained. Rather, it is necessary to confirm the subject’s intentions each time, according to the subject’s condition. As informed consent is a process of communication between researchers and study participants, it would be insufficient for it to be obtained after an explanation is provided only once. Therefore, at each stage of research, it is desirable that appropriate explanations are provided, according to the research participants’ capacity to understand, so that sufficient understanding can be obtained. For example, in dementia, mental capacity fluctuates over time. In addition, patients with psychiatric disorders are more likely to change their minds once they have given consent due to psychological symptoms such as anxiety. Therefore, even if consent is obtained at the beginning of the research introduction, feelings may change on the day of the actual testing. In addition, if the study involves multiple time-course measurements, even if consent was obtained for all tests included in the plan at the beginning, the patient's condition, including his or her ability to make decisions, may change over time. Therefore, it is necessary to confirm the subject’s will at the appropriate time by verbally confirming consent again before the tests and by taking the patient’s dissent into consideration.

When conducting research involving patients who have lost the capacity to make decisions regarding participation in research, consent from a surrogate is often required. Even in situations where consent for participation in research is given by a surrogate, it is desirable that as much explanation as possible be provided to the research participant him/herself from the perspective of relational autonomy. When providing consent seems cumbersome for the research participant, the individual must be provided with an explanation in accordance with their understanding, even if their capacity to provide consent is insufficient by itself, as long as it is not entirely absent. One way to do so is to meticulously create explanatory documents. In other words, an assent will then be required. Assent is often referred to in the case of minors. However, explanations must also be provided in the case of individuals with vulnerabilities, that is, individuals whose capacity for judgment is impaired for whatever reason.

Simultaneously, dissent is an important concept in that assurance of “withdrawability of consent” has an important place in informed consent for research participation. In research on individuals with vulnerabilities, special consideration regarding dissent is required from the viewpoint of the protection of vulnerable individuals, such as the presumption of intent to refuse, which is deduced from simple gestures and facial expressions when such participants have difficulties expressing their intentions verbally. The rationale for respecting the person’s refusal to participate with this level of care is sought due to the fact that patients with psychiatric and neurological disorders are entities that can achieve relational autonomy with the support of their surroundings.

#### Relational Autonomy

Relational autonomy assumes that for individuals to be autonomous, support from those around them is necessary; the ability to establish such supportive relationships is a mark of an individual’s autonomy (Christman and Anderson 2008; Mackenzie and Stoljar 2000; Mackenzie 2008). Therefore, rather than concluding that autonomy is an individual issue, interpreting it as a relational issue between individuals and those around them could lead to the following understanding: autonomy for individuals with psychiatric or neurological disorders, who need support from daily caregivers, depends on their actual life circumstances.

From the concept of relational autonomy, it is desirable to obtain consent from participants with vulnerabilities, based on their own determination of study participation with the support of researchers and medical professionals, rather than obtaining informed consent on a casual basis from the guardians or proxies of vulnerable participants who lack the capacity to provide consent (3–5). Allowing vulnerable subjects who partially lack the capacity to consent to make their own decisions about participating in research, supported by researchers and medical professionals, might be seen as paternalistic behavior from a strict concept of autonomy. However, relational autonomy ethically justifies the decision of vulnerable subjects to participate in research on their own through the support provided by researchers and medical professionals, which is seen as respect for autonomy.

In other words, consideration for respecting the research participant’s relational autonomy is necessary, based on support from the guardian or proxy, rather than allowing the study participant to engage in the research based solely on proxy consent, because the research participant’s judgment alone is insufficient. With this in mind, the following needs should be considered: (1) scrutiny of whether the case truly requires consent and (2) the selection of proxies for those who are able to understand the psychological nature of the research participant and his or her individual personality for comprehending the humanity of the research participant and maximize the individual’s interests. It is not the simple choice between consent from the individual concerned or from a proxy that is important, but rather that vulnerable individuals, such as those with psychiatric or neurological disorders, can make gradual progress towards autonomy with the support of their proxy. Moreover, it is desirable to build a virtuous cycle to support the research participation of vulnerable participants, by which the proxy can grow to become better representatives, and the individual’s intention can thus be grasped more accurately based on the proxy’s support. Physicians, nurses, and other healthcare professionals, as well as medical and other researchers, are expected to play a mediating role in collaborative relational autonomy efforts that involve vulnerable research participants or participant candidates with their proxies. Generally, it is not desirable for the attending physicians, nurses, and other healthcare professionals to be involved in informed consent in research, from the perspective of coercion. However, from the perspective of relational autonomy, it is desirable for physicians, nurses, and other healthcare professionals who are familiar with the patient’s condition and medical status to actively participate in the explanation and provide support, especially in the contexts of highly invasive research.

#### Special Considerations

From the perspective of vulnerabilities, special considerations are required when conducting research with vulnerable individuals—such as when individuals have difficulties expressing intentions verbally, which would require the frequent confirmation of the presence or absence of adverse events (Binik and Weijer 2014). Other special considerations are necessary to ensure that individuals with vulnerabilities can obtain appropriate care even after the research is concluded, such as through the establishment of a contact office. Further, from the perspective of the right to know, research results need to be communicated in an appropriate manner (4–1).

Consideration of the family, acquaintances, and other relevant parties is also due, which is a unique requirement for individuals with vulnerabilities. Such actions are necessary because family members, acquaintances, and other relevant parties are positioned to support the relational autonomy of vulnerable research participants. From the perspective of substantial guarantees of the aforementioned right to know, access to the publicized research results should be granted not only to the research participants but also to their family, acquaintances, and other relevant parties (4–2).

Furthermore, regarding research that involves vulnerable individuals, efforts are required (whenever possible) from the perspective of feedback for future research. Efforts to enable improvements in future research are necessary, including interviews with research participants and related parties, such as families and acquaintances, on the following topics: any anxiety that participants may have experienced after participating in the research (e.g., sudden changes in their physical condition) and the type of satisfaction participants experienced (e.g., satisfaction regarding societal contributions and altruistic acts) during or after the research.

## Example Case

A patient with schizophrenia who has no relatives received an announcement about a research project from his doctor. The patient came to the researcher for an explanation of the study, accompanied by a friendly neighbor who usually takes care of him in various ways.

This study is to examine brain regions and functional abnormalities assumed to be associated with negative symptoms of schizophrenia, and the only way to obtain valid results is to use schizophrenic patients as subjects. If the areas and functional abnormalities associated with negative symptoms are confirmed, it may lead to drug discovery for negative symptoms of schizophrenia, which may be in the public’s interest. Therefore, it is ethically acceptable to conduct research on patients with schizophrenic, even if it imposes a double burden on the subjects.

Because the patient was not fully competent to make decisions in terms of mental capacity, the researcher prepared an explanatory document that was easier to understand than usual and explained the study to the patient and his accompanying friend who explained this point to the patient. However, the patient wanted to participate because he wanted to help everyone. Since the friend accompanied the patient to his usual outpatient clinic, he brought up the example of B and C, who were also patients with schizophrenia and whom the patient knew. He asked the patient if he wanted to be helpful to B and C, even if the study was not helpful to himself, to which the patient replied, "because that would make me happier.” Although the patient did not have the capacity to consent to participation in the study by himself, he exhibited relational autonomy with the help of his friend and researcher. Additionally, the patient is consenting to participate in the study of his or her own will, rather than participating in the study through a friend's proxy presumption.

In order to ensure the patient's relational autonomy, the researcher should explain the research carefully to the friend as well as the patient and describe how to ask for assistance and withdraw from participation. Furthermore, the researcher should instruct the investigator to consider stopping the test once the patient shows signs of not wanting to participate in the test during the course of it. This is to ensure that a framework is in place to allow informed descent.

## Conclusion

The abovementioned guidelines touch upon the special considerations that are necessary for improving research involving participants with psychiatric and neurological disorders. It is based on the central concepts of “vulnerabilities,” “relational autonomy,” and “dual burden” The central concepts can be organized in relation to the three principles of research ethics set forth in the Belmont Report as follows. Emphasis on relational autonomy, consideration for vulnerability, and reduction of dual burden each correspond to the specific considerations required by the preceding general principles, when the general principles of research ethics of respect for personality (respect for autonomy), good conduct, and justice are applied to research involving patients with mental and neurological disorders. The uniqueness of these guidelines lies in the fact that the idealistic principles of the Belmont Report are not applied directly to research on patients with mental illness but are adjusted and re-presented in a form that is practically usable.

Nevertheless, the guidelines represent a proposal for highlighting the general direction of progress, through the case-by-case measures that are required, depending on the characteristics of each psychiatric or neurological disorder in question, and the details of the specific plans for research on such issues.

### Supplementary Information

ESM 1 (DOCX 96.2 kb)
