# Supplementary material for: Ethics Guideline Development for Neuroscience Research involving Patients with Mental Illness in Japan
Source: Asian Bioeth Rev. 2023 Feb 10;15(4):365–75. doi: 10.1007/s41649-023-00240-x (PMC10555971; doi:10.1007/s41649-023-00240-x)
Supplement: Supplementary file 1 — (DOCX 96.2 kb) [file 41649_2023_240_MOESM1_ESM.docx]

Appendix

**Ethical Guide for Research on Mental and Neurological Disorders**

**(Revised edition)**

November 2019

Strategic Promotion Program for Brain Science Research

“Research on resolving ethical, legal, and social issues in brain science research”

**Table of Contents**

**Introduction: Why Are Special Ethical Considerations Necessary?**

1. **A Researcher's Guide to Better Study Design**
2. **Selection of Research Participants**
3. **Risk-benefit Assessment**

**2-1. Minimal Risk**

**2-2. Risk Minimization**

**2-3. Benefit Maximization**

1. **Informed Consent**

**3-1. Background**

**3-2. Measurement of Consent Capacity**

**3-3. Consent by the Individual**

**3-4. Assent and Dissent**

**3-5. Consent by a Legally Acceptable Representative**

**3-6. Privacy Protection**

1. **Special Considerations for Research Participation**

**4-1. Considerations for Research Participants**

**4-2. Considerations for Family, Friends, etc.**

1. **A Guide to an Appropriate Ethical Review System**
2. **Appropriate Ethical Review**

**5-1. The Nature of the Ethics Review Committee**

**5-2. Research Ethics Support for Researchers**

**5-3. Compliance with the Clinical Trials Act**

**Conclusion**

**Introduction: Why Are Special Ethical Considerations Necessary?**

This "Ethical Guide for **Research on Mental and Neurological Disorders**" (draft), as the title suggests, is an "ethical guide" that outlines important points to be considered in order to make research involving patients with mental and neurological disorders more ethically appropriate. We have prepared this guide so that you can have a quick and systematic understanding of the ethical points that should be noted and taken into consideration.

**Part A** presents key points for researchers on how to **design ethically appropriate research**, while **Part B** offers recommendations on how to **improve the ethical review process.**

Some of you may already have questions about why special ethical considerations are necessary for patients with mental or neurological disorders, or why they need different procedures. We would like to start the discussion there.

To begin with, medical research involving human subjects requires different considerations from other types of medical research. This is because the welfare or rights of the research participants must not be compromised or infringed upon in the course of the research. (For non-human subjects, such as drugs, "welfare" and "rights" are not discussed, but for human subjects, welfare and rights are very important. If we overlook this distinction, we may end up treating people like objects.) The preamble of the “Ethical Guidelines for Medical and Health Research Involving Human Subjects” states that “medical and health research involving human subjects can have a major impact on such research subjects both physically and mentally or, indeed, on society itself, as well as cause a variety of ethical, legal, or social problems. The welfare of those research subjects shall be given priority over scientific and social results of research, and human dignity and rights shall be protected.”

Specifically, three major considerations must be made: **informed consent to respect the personality of the** **research participant**, **risk/benefit assessment to ensure benefits to the research participant**, and **appropriate selection of research participants to conduct the research in a just manner** (Belmont Report). Institutionally, an **Ethics Review Committee** is established to check whether the above considerations have been adequately made.

The same considerations must be made for research on mental and neurological disorders. However, in the case of these disorders, there are significant difficulties with respect to “consent” in informed consent to respect the personality of the research participant himself/herself. This is crucial indeed. Strict adherence to the **principle that research participation requires the appropriate consent of the participant** would preclude any research participation if the patient lacks the capacity to consent. However, if research participation is not permitted at all, research on these disorders will never progress, and this will be a great loss to society as a whole. However, if we simply say that research can be conducted without consent in the case of these disorders, we may not be able to properly protect the welfare and rights of the research participants.

Thus, in the case of research on mental and neurological disorders, there is a sharper ethical dilemma between the protection of research participants and the interests of medical research than in ordinary medical research. ^[[1]](#footnote-1)^

Therefore, research on disorders that involve deficits in the capacity to consent must be justified by other criteria than the usual approach. In other words, **special care must be taken in conducting research to ensure that there is no unjustified loss to the individual, even without his or her consent**. This is why **special ethical considerations** are required for patients with mental or neurological disorders.

Characteristics that **require special consideration in** research participation, such as mental or neurological disorders, are called vulnerabilities. Vulnerability, as discussed above, can be viewed primarily as an impaired decision-making capacity, but there are many other ways to think about it. This Ethics Guide briefly presents the desirability of taking vulnerability into consideration.

**Ethical point 1: Vulnerability**

There is a wide range of ethical discussion on vulnerability. (1) Vulnerability is not the same as disease (Dukoff & Sunderland 1997). Even if a person suffers from a disease that affects cognitive abilities, there are varying degrees of impact. At the stage when the person is affected but still has the capacity to consent, the person's consent (or the selection of a legal representative) may be acceptable. (2) Vulnerability is not the same as cognitive capacity deficits (Levin et al. 2004, Goodwin 2016). In addition to deficits in capacity to consent, there can be cases in which socioeconomic contexts (environment and power relations) make people more vulnerable to harm. For example, the history of black people in the U.S. being unfairly forced to participate in research falls under this issue (although the widely referenced CIOMS guideline states that vulnerability is the lack of capacity to consent). (3) Vulnerability is not always a group characteristic (Levin et al. 2004). Not only group characteristics including a particular disease or race, but also personal circumstances are subject to special consideration. Examples can include pregnancy, allergies, PTSD, etc.

Thus, the exact level of consideration required will vary from case to case. Nevertheless, we hope that this guide will be sufficient for you to grasp the basic idea.

**A. A Researcher’s Guide to Better Research Design**

1. **Selection of Research Participants**

The basic principle of “selection of research participants” in research on mental and neurological disorders is that the following: Research that can be performed on **other research participants should not be conducted on patients with these disorders**. In other words, research that **can be performed on non-vulnerable persons should not be performed on vulnerable persons**. Research should not impose unnecessary risks on vulnerable persons. Historically, there have been instances of unjustifiable participation in research because it is easier to recruit mentally ill or neurologically ill patients^[[2]](#footnote-2)^. This is not acceptable today.

However, this does not mean that vulnerable persons are not allowed to participate in research at all. As noted in the Introduction to this guide, it would be a problem if no finding on mental and neurological disorders is to be obtained at all. There is nothing wrong with selecting vulnerable persons as research participants if the purpose is to obtain findings that can only be obtained with vulnerable persons.

A troubling case: In reality, there may be cases in which a patient with some capacity to consent clearly has, or once had, a strong desire to participate in research in order to contribute to society, , and the family wishes to respect it. In such cases, it may seem unethical to refuse the patient’s participation in the research on the grounds that his/her participation is not necessary (i.e., the research can be conducted on non-vulnerable persons), and thus not respect his/her wishes.

This is a difficult issue, but if the research can be conducted on people with no vulnerability, we **should avoid placing unnecessary risks on vulnerable individuals**. This is because if exceptions are made to allow participation in research on the basis of “strong will of the patient”, there is a risk that such instances will increase dramatically.

In the first place, it is important to note in medical research that the **advancement of research on a person’s disorder is separate from his or her therapeutic benefit** (social benefit ≠ personal benefit). This is because participation in research does not necessarily benefit the participant (and it is precisely because there is no guarantee of a benefit that necessitates research). The decision on research participation must be based on the above basic principles and also on the **risks and benefits** to the individual concerned. ^[[3]](#footnote-3)^

So, let’s now look at the risk-benefit assessment.

1. **Risk and Benefit Assessment**

**2-1. Minimal Risk**

It is useful to distinguish between the “**definition**” and the “**operation**” of the concept of “**minimal risk**.” Although it is desirable to keep the risk below minimal, the basic idea is that research that exceeds minimal risk may be acceptable when weighed against the social significance of the research. It is important to define minimal risk unambiguously, and then consider what conditions must be met for the research plan to be approved, when it is expected to exceed the minimal risk.

First, **minimal risk is defined as the degree of risk imposed in daily life.** Specifically, it corresponds to the degree of invasiveness associated with **medical checkups and routine examinations**. The categories of risk that exceed minimal risk can be classified as “slightly more than minimal risk (minor increase)” and “greater than minimal risk (greater than minor increase). The following is a rough guide to these risk categories:^[[4]](#footnote-4)^ . It would be unrealistic to define unambiguously which of these categories each intervention in a study falls into. For example, while the (objective) degree of invasiveness of an intervention may be defined, the possible adverse effects of that intervention may well be on a case-by-case basis, depending on the condition of the study participants (patients with mental and neurological disorders). A careful case-by-case risk assessment, taking into account the vulnerability of patients with mental and neurological disorders, is the preferred approach. As a rough guide, one might consider the following.

- Minimal risk: top-up blood draws, etc.
- Slightly above minimal risk: MRI + sedation, novel therapies, questions about abuse experience, etc. (psychological invasion).
- Considerably above minimal risk: washout, with control group, case observation to see triggering factors.

In general, **the greater the risk, the more stringent the requirements for research to be considered ethically valid**.

In “principle” the risks involved in a research plan should be within the “minimal risk” range. Conditions for approval of a research plan that exceeds minimal risk include the following: there are no other alternatives than using patients with mental or neurological disorders as research participants (see “Selection of Research Participants” above), the research plan is highly achievable, the social benefits of achieving the research plan are large, and the risks and benefits are well balanced (see “2-2. Risk Minimization” and “2-3. Benefit Maximization”).

2-2. R**isk Minimization**

We are not saying that research that imposes risk in excess of minimal risk is acceptable because much research exceeds minimal risk, or, in other words, because many studies would not be possible if minimal risk were strictly applied as defined. Let us step away from the context of research and consider the nature of risk-bearing in our daily lives. A classic example of taking on more risk than we would normally take on in our daily lives is **altruistic behavior**. For example, when we try to rescue a drowning person in the ocean or a person who has fallen off a train platform, we are assuming a certain probability of injury or death. Such a risk is clearly beyond the degree imposed in our daily lives. Yet such risk-bearing is acceptable because we **voluntarily assume such risks** in altruistic acts that seek to protect the interests of others rather than ourselves. The argument can be summarized as follows: **if the two requirements of altruistic motive and voluntariness are satisfied, it is permissible to assume more risk than is imposed in everyday life.** Under these two requirements, the degree of acceptability of various risks cannot be defined unambiguously but it is determined on a case-by-case basis according to social norms while taking into consideration the balance with their associated benefits.

The same applies to research that involves risks beyond those imposed in everyday life. As discussed below (see “Ethical Point 3: Distinction Between Treatment and Research”), research is conducted to obtain generalizable knowledge, and it is uncertain whether there will be any direct therapeutic benefit to the research participants. Then, participating in research can be considered a kind of altruistic act when it is not necessarily for one’s own benefit but in the hope that it will improve diagnosis, treatment, and prevention for the benefit of patients and future patients suffering from the same disorder. Of course, in the **case of patients with mental or neurological disorders**, even if the altruistic motive requirement is satisfied, there is still the issue of judgmental capacity, so it must be carefully assessed **whether they are voluntarily accepting risks beyond those imposed in their daily lives** (for example, whether there is inducement or manipulation in the decision to assume risk).

**Minimizing risk** is the principle that when the risks associated with a study exceed minimal risk, **the risks should be as small as possible when weighed against the expected benefits,** regardless of whether the participants are vulnerable or not. While the greater the expected benefit, the greater the acceptable risk, a more careful risk assessment is essential in the case of research participation by patients with mental or neurological disorders. This is because there is a **concern that unexpected adverse events may be more likely to occur than in the case of healthy participants.**

For example, let us consider the case of a patient with a mental or neurological disorder who participates in a study while he or she visits a hospital. If the side effects associated with the new treatment or drug included in the study occur after the patient returns home, and the patient lives alone, it is possible to imagine a scenario in which the patient is unable to call an ambulance and go to the hospital and falls into a serious condition (unlikely but worst-case scenario). **The probability and severity of risk may be different** for patients with mental or neurological disorders than for healthy participants, depending on factors such as the presence or absence of a roommate and the ability to cope in case of emergency.

This is closely related to the question of what aspects of eligibility should be considered when selecting research participants. The risk presented by the researcher (objective/quantitative risk) may differ from the risk perceived by the research participant (subjective risk: perception/interpretation of objective/quantitative risk). A 20% risk (from the researcher’s perspective) that a treatment will cause side effects is quantitative, but how seriously that 20% risk is perceived by the research participant is subjective. Researchers tend to view risks quantitatively, whereas research participants tend to take the numbers more seriously than they really are. In light of this, the principle of “minimizing risk” in research participation becomes even more important for patients with mental and neurological disorders than for healthy research participants because of the “double burden” (Ethical Point 2). So what specific measures are needed to minimize risk?

First, **the risk assessment should be done carefully**. The same risk (e.g., itchiness or pain felt while wearing some device) may not be communicated to the researcher by patients with certain mental or neurological disorders. Including considerations for such “vulnerabilities” in the research design will result in an appropriate research design that minimizes risk.

Second, **it guarantees the freedom to withdraw consent to participate in research, i.e., the “freedom to withdraw” from the research.**^[[5]](#footnote-5)^ If something goes wrong during research, it is not always possible for patients with mental or neurological disorders to accurately express their wishes or for family members or other legally acceptable representatives to always have an accurate picture of what is happening. Depending on the situation, the researcher may need to be flexible, for example, to discontinue participation in the study at his or her discretion. When vulnerable research participants are involved, it is also extremely important in the mid- to long-term to have the courage to stop the research if it is deemed unsafe, in order to avoid undermining public trust in the research.

Let us explain a little more about the careful assessment of risk. When assessing risk, it is necessary to look at both the degree (severity) of risk and the probability (frequency) of risk occurrence. Distinctions can be made between mild/moderate/severe for severity and low/medium/high for frequency of occurrence^[[6]](#footnote-6)^. Combining these distinctions, risks can be classified into nine categories. Depending on which category it falls into, you may or may not be able to cope with it and under what conditions the study is allowed in the first place **(see Chart 1 and Chart 2)**.

The reference Charts 1 and 2 are tentative drafts to be used as a starting point for discussion, and we would like to elaborate the framework for risk assessment from an ethical perspective in line with actual situations in the field by receiving information on ethical issues and difficult cases faced by researchers who are actually engaged in research.

**Ethical Point 2: Double Burden**

It is called "**double burden"** when **those who have previously borne more of the burden than others are treated even more unfavorably.** This is effectively "whipping the weak," and from an ethical standpoint, it is a practice that should be particularly avoided. It occurs when (1) a system does not take into account the degree of burden that people had to bear before and (2) it further disadvantages those who had to bear much. It can be said that, **from the viewpoint of double burden, patients with mental or neurological disorders** who are already under a certain burden **should not be subjected to excessive risk in participating in research.**

In light of the above, the risks of participating in research are different for patients with mental and neurological disorders and healthy participants, in terms of how serious the risks can be and whether they can cause unanticipated events, as already mentioned. In the case of vulnerable research participants, such as patients with mental or neurological disorders, risk assessment requires a more detailed evaluation of the potential impact of research participation on their living conditions than is required for healthy participants. Their living conditions here include not only physical and mental health, but also the ability to work and participate in society.

In philosophy and ethics, the terms “welfare” and “well-being” are used to refer to the quality of living conditions. If a person’s level of welfare or well-being is sufficient, he or she is considered happy. However, what should we focus on when evaluating a person’s welfare or wellbeing? Based on research in philosophy and ethics on welfare and well-being^[[7]](#footnote-7)^ and putting it into the context of research participation, the following factors can be relevant.

1. Smooth daily living: Are the participant’s health status, activities of daily living, and fulfillment of biological needs sufficient for smooth daily living?
2. Smooth social life: Is the participant able to lead a smooth social life in terms of interpersonal relationships and social participation?
3. Mental and physical integrity: Are the participant’s physical and cognitive functions working properly as a single person?
4. Happiness (self-esteem, self-evaluation): To what degree is the participant satisfied with his/her life as a whole, including whether or not he/she has a purpose in life.

At first glance, these include some factors that would not be an issue in normal clinical research. However, (1), (2), and (3) relate to direct therapeutic benefits to the research participants, and (4) relates to indirect benefits in the form of fulfillment through social contribution by research participation. Let us now turn to this issue of benefits.

**2-3. Benefit Maximization**

When vulnerable participants, such as patients with mental or neurological disorders, must be included in a research plan, the first step is to keep the risks imposed on the participants below minimal risk, and if this is not possible, the portion exceeding minimal risk must be as small as possible. Now, as mentioned in “2-2.”, **since risks are evaluated in relation to benefits, in addition to minimizing risks,** another principle required for research is to **make the benefits as large as possible.**

Note that the meaning of benefits differs between treatment and research. Focusing on the “distinction between treatment and research,” clinical research aims to “**acquire generalizable knowledge,**” whereas treatment aims to provide medical benefits to patients. Also, in research, there is uncertainty as to whether the patient can have any direct medical benefit by participating in research. When evaluating benefits, it is useful to consider their diversity and categorize them as follows:^[[8]](#footnote-8)^ .

- (1) Benefits to study participants: (1-a) direct benefits, (1-b) indirect benefits
- (2) Benefits to others (social benefits)

**Direct benefits** are cases in which research participants are the beneficiaries of medical care, such as being cured of a disease or having their symptoms improved through participation in research. With regard to indirect benefits, the question often arises as to whether to pay honoraria and transportation costs for participation in research. These are not limited to vulnerable research participants and not discussed in detail here.

However, what is different from conventional research is that vulnerable research participants may benefit from **access to enhanced professional care through participation in research,** even if there is no direct benefit to them. Another **indirect benefit** is **the psychological fulfillment that comes from the altruistic activity and social contribution** of participating in research that may lead to the development of treatments for future patients, even if there is no benefit to oneself. Nevertheless, it is unclear whether a patient with a relatively severe mental or neurological disorder would be able to feel such a benefit. It is also undesirable to imply such possible indirect benefits to potential research participants, i.e., patients with mental or neurological disorders and their families, in order to induce them to participate in the study.

**Benefits to others (social benefits)** include benefits to people other than the patients themselves, specifically, their families, care providers, patients suffering from the same disorder, and future patients who may suffer from the same disorder. The improvement in the patient’s condition that results from participation in the study, however slight, will reduce the burden on family members and care providers, and the discovery of effective treatments will be of great benefit not only to the patient, but also to patients who are currently suffering from the same disorder, and to future patients who may suffer from the same disorder.

The key things to remember about risk-benefit assessment are the following. **There is an asymmetry of benefits and risks, with different beneficiaries and risk bearers**. In research where the primary goal is to gain knowledge, it is inevitable that the beneficiaries are the future patients and society in general, while the risk bearers are exclusively the research participants. When research participants are vulnerable, the above points require more careful consideration and handling than in normal research. It is easy to imagine that if one were to weigh the risks to a single research participant against the benefits to a large number of beneficiaries, the balance would tip in favor of the latter. However, no matter how great the social benefits, we must avoid disregarding the dignity of the individual. From the standpoint of human dignity, certain limits should naturally be set on the risks imposed on individuals. This requires a careful risk-benefit assessment.

When obtaining **Informed Consent (IC) for participation in research,** it is advisable to carefully explain to the research participant or legally acceptable representative that **any of these benefits are probable and never promised**. It is also important for them to understand that **the research is basically intended to lead to the development of future treatments and is not intended to provide direct medical benefits,** so as to avoid “**misunderstanding between treatment and research**” (Ethics Point (3)) before they participate in the research.

Please note that appropriate and effective clinical research can only be promoted when three factors are in place: the **professionalism of the researchers**, the **altruism and goodwill of the research participants**, and the **trust of society in the research.**

**Ethics Point 3: Misunderstanding Treatment and Research**

　　Although there is essentially no clear benefit to the participant in participating in research, research participants often mistakenly believe that participation in research is a beneficial "treatment" (e.g., access to the latest treatments). Or they may believe that the treatment provided in research is tailored to the individual needs of the participant (rather than to acquire general knowledge). This misconception is called the "therapeutic misconception" (Appelbaum et al. 1982).

　　If there is a misunderstanding between treatment and research, research participants and their legal representatives may voluntarily accept excessive risk by overestimating the benefits. When obtaining consent for participation in research, it is necessary to make sure that people understand not only the risks and benefits, but also that this is a study and not a treatment in and of itself.

**Chart 1: Guideline of Required Responses, depending on the Degree and Frequency of Risk**

| **Degree**  **Frequency** | **Mild** | **Moderate** | **Severe** |
| --- | --- | --- | --- |
| **Low Probability**  **(unlikely)** | **Discomfort and pressure from the device ⇒ Special consideration is required because of the possibility of health hazards and inducing disturbing behavior.** | **Temporary worsening of the disorder due to placebo or washout => Action may be required, such as halting the study or providing treatment.** | **Severe worsening of the disorder due to placebo or washout ⇒ Depending on the situation, action may be required, including discontinuation of the study.** |
| **Medium Probability**  **(possible)** | **Discomfort and pressure from the device ⇒ Special consideration is required, regardless of the degree of health damage, as the person may not be able to express his/her will.** | **Temporary worsening of the disorder due to placebo or washout => Action may be required, such as halting the study or providing treatment.** | **Severe worsening of the disorder due to placebo or washout ⇒ Depending on the situation, action may be required, including discontinuation of the study.** |
| **High Probability**  **(probable)** | **Discomfort and pressure from the device ⇒ Special consideration for monitoring, etc. is required even without serious health damage.** | **Temporary worsening of the disorder due to placebo or washout => Action may be required, such as halting the study or providing treatment.** | **Severe worsening of the disorder due to placebo or washout ⇒ It is difficult to justify conducting the study.** |

The above is one guideline for considerations and response policies. Careful consideration and response should be made on a case-by-case basis, depending on the intervention method and the condition of the research participant.

**Chart 2: Ethical and Overall Assessment Guideline for the Degree and Frequency of Risk**

| **Degree**  **Frequency** | **Mild** | **Moderate** | **Severe** |
| --- | --- | --- | --- |
| **Low Probability**  **(unlikely)** | **Minimal risk** | **Slightly above minimal risk => Additional protective measures such as monitoring are required.** | **Considerably above minimal risk ⇒ If there is a possible therapeutic benefit to the person, it should be conducted in the presence of a health care professional.** |
| **Medium Probability**  **(possible)** | **Minimal risk but caution is required, depending on the condition of the participant ⇒ additional protective measures such as monitoring are required.** | **Slightly above minimal risk ⇒ Additional measures such as monitoring are required and should be conducted in the presence of a healthcare professional.** | **Considerably above minimal risk ⇒ If there is a possible therapeutic benefit to the person, it should be conducted in the presence of a health care professional.** |
| **High Probability**  **(probable)** | **Minimal risk but caution is required, depending on the condition of the participant ⇒ additional protective measures such as monitoring are required.** | **Slightly above minimal risk ⇒ Additional measures such as monitoring are required and should be conducted in the presence of a healthcare professional.** | **The risks clearly outweigh the benefits, making it difficult to justify conducting the study.** |

The above is one guideline for risk assessment and response policies. Careful consideration and response should be made on a case-by-case basis, depending on the intervention method and the condition of the research participant.

1. **Informed Consent**

**3-1. Background**

The ideological background of the **principle of respect for personality** is the philosophy of Immanuel Kant, an 18^th^ century German (then Prussian) philosopher whose influence continues to be felt today. Kant argued that reason commands us, as an ethical imperative, to act in such a way that you treat humanity, whether in your own person or in the person of any other, never merely as a means to an end but always at the same time as an end. The ultimate goal of brain function research is, of course, to elucidate human brain function, and research may be conducted on animals occasionally for this purpose, but research must be conducted on humans after all. In this respect, we are forced to use humans as **a means for our research**. What is problematic from the perspective of Kantian philosophy is brain science research that **uses people “only” as a means**. Such brain science research is extremely difficult to justify by Kant’s ethics.

So, in what cases can we deny that humans are used “only” as a means to an end? It is when the research is conducted with the **voluntary consent of the research participants**. The emphasis must be placed on “voluntary” here. When a research participant voluntarily wishes to participate in research out of his or her own conscience, hoping that his or her participation will advance the knowledge of humanity through the progress of our understanding of brain function and the happiness that will be achieved by that knowledge, the **research participant is not being used only as a means to an end, but he or she is being used as an end at the same time.**

Now that we have presented the major principle of “respect for autonomy” in biomedical ethics, autonomy takes on a special meaning in the context of the participation of patients with mental and neurological disorders in research. Autonomy is not a concept that can be viewed in terms of all or nothing, or whether it exists or not, as if the person’s autonomy can be ignored because he or she is incapable of making judgements. Rather, this guide bases its discussion on the concept of **relational autonomy^[[9]](#footnote-9)^** as a guiding thread in dealing with the specific and challenging case of patients with mental or neurological disorders.

Relational autonomy is the idea that **individuals need support from their surroundings in order to be autonomous, and that being able to form such supportive relationships is what constitutes individual autonomy**. Thus, viewing autonomy not as an individual problem but as a problem of the relationship between the individual and the surrounding environment leads to an **understanding of the autonomy of patients with mental and neurological disorders who need the support of care providers on a daily basis, in line with their actual living conditions.** Care providers here are not limited to family members and friends, but also include physicians, nurses, and other health care professionals. The key point of the concept of relational autonomy is to support the autonomy of research participants not only from the person himself/herself but also from those around him/her, such as family, friends, medical professionals, and medical researchers. If we adopt this perspective of relational autonomy, patients with mental and neurological disorders can also be autonomous with the support of their surroundings. Supporting autonomy in this sense is an issue that should be addressed not only in medicine and medical research, but also in society at large. In the sections below, we discuss the following issues related to informed consent: measurement of capacity to consent, consent by the individual, assent and dissent (see below), and consent by a legally acceptable representative. The importance of these procedures for informed consent is based on the **principle of relational autonomy to respect patients with mental and neurological disorders as individuals**.

**3-2. Measurement of Consent Capacity**

　The first step required in the informed consent process is to ascertain the capacity of the research participant to consent. Capacity to consent can be divided into **legal capacity to consent (legal capacity to respond)** and **psychological capacity to consent.** Consent from a psychological perspective presupposes that the participant has the capacity to make a judgment. Legal capacity to consent, on the other hand, adds age and other conditions in addition to judgmental capacity. In research on mental and neurological disorders, the assessment of judgmental capacity is important to determine whether a research participant is capable, by himself/herself, of giving informed consent to participate in research.

The construct of judgmental capacity has changed over time, but the one currently in widespread use was proposed by Appelbaum & Grisso. It consists of the four components of **“understanding,” “recognition,” “rationality,” and “statement of intent.” Understanding** refers to the ability to **understand information about research**. Recognition means being able to **relate the information to oneself** (e.g., being able to recognize the risks associated with the research as something that can happen to oneself). Rationality means being able to **process information rationally** (e.g., making an overall judgement to participate in the research, while feeling a little scared, because of eagerness to contribute to the understanding of this disorder). Statement of intent means that you are able to communicate your intentions (not necessarily verbally).

In light of this construct, we evaluate the subject’s ability to make judgments. At this point, the subject’s capacity for judgment is not evaluated as a binary choice between “yes” and “no”. The **ability to consent to research participation is determined by the relative relationship between the judgmental capacity of the research participant and the judgmental capacity required for research participation.** In a medical situation, the capacity to make judgements is also determined by the relative relationship with medical treatment, but because **there is usually no benefit from treatment in research, the capacity to make judgements required is set higher than in medical treatment** (e.g., the capacity to make judgements required for a PET scan for research is higher than that required for a PET scan for medical treatment).

**3-3. Consent by the Individual**

When the research participant has the capacity to consent as explained in 3-2, consent will be obtained from the research participant him/herself. The “Ethical Guidelines for Medical and Health Research Involving Human Subjects" (enacted in 2014, revised in 2017) requires the following 21 items to be explained to research subjects with regard to informed consent. Some types of research do not necessarily fall under this category, and some types of research also do not necessarily require written explanation and consent or prior opt-in consent, but basically, efforts should be made to explain these 21 items.

(1) Title of the research and the fact that approval of the chief executive of the research implementing entity has been given concerning its implementation;

(2) Names of the research implementing entity and the principal investigator (including names of the collaborative research implementing entity(s) and principal investigators of such collaborative research implementing entity(s), when the research is conducted collaboratively with other research implementing entity(s));

(3) Objectives and significance of the research;

(4) Method and time period of the research (including purpose of the utilization of specimens or information acquired from the research subject);

(5) Reasons why asked to be enrolled in the research;

(6) Burdens to be caused on the research subjects and predictable risks and benefits;

(7) The fact that research subjects, etc. may withdraw their consent at any time even after they have given consent with regard that the research is commenced or continued (when it can be difficult to take measures that follow the withdrawal made by the research subject, etc., a statement to that effect and the reason for the difficulty);

(8) The fact that the refusal or withdrawal of consent by a research subject, etc. with regard that the research is to be commenced or continued does not cause any disadvantage to such research subject, etc.;

(9) Means to make information on the research public;

(10) The fact that research subjects, etc. can request and obtain or read the research protocol and documents concerning method of the research, to the extent it does not interfere the protection of personal information, etc. of other research subjects, etc. or the originality of the research, as well as the procedure to obtain or read such protocols and documents;

(11) Handling of personal information, etc. (including process of anonymization, when anonymization is conducted);

(12) Means for storage and disposal of specimens and information;

(13) Status of research-related conflicts of interest of the research implementing entity, such as research fund resources, as well as research-related conflicts of interest of each investigator, etc., such as his/her individual income;

(14) Response to consultation, etc. made by research subjects, etc. and other individuals concerned;

(15) When the research involves any financial expenditure on or remuneration for the research subject, etc., a statement to that effect and details of such;

(16) When the research involves any medical technique beyond usual medical practice, description of alternative procedure(s) or course(s) of treatment;

(17) When the research involves any medical technique beyond usual medical practice, response related to the healthcare delivery to the research subjects after the research;

(18) When any significant finding concerning the subject’s health or generic characteristics which may be inherited by his/her offspring, etc. may be obtained through implementing the research, handling of the research results related to the research subject (including incidental findings);

(19) When the research involves any invasiveness, whether or not compensation will be offered for research-related injury and details of such compensation;

(20) With respect to specimens and information acquired from the research subject, when any of those may be utilized or provided to other research implementing entity(s) for the research in future that is not identified at the time of obtaining consent from the research subject, etc., a statement to that effect and the contents of utilization assumed at the time of obtaining consent; or

(21) When the research involves any invasiveness (not including minor invasiveness) and intervention, the fact that the monitor(s), the auditor(s) and the ethical review committee will be granted direct access to the specimens and information acquired from the research subject, without violating confidentiality of the research subjects, to the extent necessary.

Furthermore, we need to recognize that **informed consent is a process, not an event**. It is not appropriate for informed consent to be used as a tool to absolve researchers. Consent based on inadequate explanation by the researcher, and the subsequent inadequate understanding by the research participant, is detrimental to the ethical appropriateness of the research. **Informed consent is** a **process of communication between the researcher and the research participant**. It is desirable that appropriate explanations be provided to research participants at each stage of the research process, and that research participants fully understand these explanations. **The pursuit of full communication is the key to respecting the research participant as a person and to conducting ethically appropriate research**.

Now, we would like to reiterate the significance of the concept of relational autonomy in terms of consent by the individual. This concept has implications, among other things, for the medical care and research participation of patients with mental and neurological disorders. Psychiatry **strives to form a rapport** between doctor and patient. It is one manifestation of trust between doctor and patient, and it is beneficial to the provision of appropriate and adequate medical care. If, however, a doctor has formed a rapport with a patient, encourages the patient to participate in research, and the patient agrees, this consent might not be totally free of the influence from the doctor-patient relationship. In such a case, a neutral third party could be present to explain the situation and obtain consent so that the patient can voluntarily consent. The involvement of such a third party to preserve the patient's autonomy is based on the idea of relational autonomy.

**3-4. Assent and Dissent**

Even if a research participant's capacity to consent is inadequate, as long as it is not zero, it is **desirable that explanations be provided to the research participant himself/herself according to his/her capacity to understand**. This is also a concept derived from the perspective of relational autonomy. When explanations are given to research participants themselves, especially to minors, this is called “**assent”**. However, this idea of assent is not only necessary for minor research participants, but also applies to adult research participants who have lost their capacity to make judgements for some reason. Even when a person's capacity to make judgements has declined, it is necessary to provide as much support as possible from the perspective of relational autonomy.

One example of such support is the creation of an explanatory consent document. The consent document should be designed to be fully understandable, depending on the individual circumstances of the research participant. Research involving minors and those who have lost the capacity to make judgements regarding participation in research often requires the consent of a legally acceptable representative. Even when consent to participate in research is given by a legally acceptable representative, it is desirable that as much explanation as possible be provided to the potential research participant, and appropriate explanations should be provided together with an easily understandable assent form for the potential research participant.

In the case of healthy research participants with the capacity to make judgements, the focus of informed consent is the possibility of withdrawing consent. If the relational autonomy of patients with mental or neurological disorders is to be taken seriously, it is desirable that explanations be given in a manner appropriate to the person's ability to understand (i.e., **assent**), and if the person exhibits behavior (including facial expressions and gestures), whether at the time of explanation or at the beginning of the research, that clearly indicates a refusal to participate, then at that point it would be **desirable to allow the option of withdrawing from the research**. This is called "**dissent**," and the rationale for respecting the individual's refusal to participate must be based on the fact that patients with mental and neurological disorders are unique individuals who can achieve relational autonomy with the support of their surroundings.

**3-5. Consent by a Legally Acceptable Representative**

In some research on mental and neurological disorders, it may be the case that potential research participants have difficulty fully understanding the research content and making an informed decision about research participation. In such cases, a consent will be taken from a legally acceptable representative. However, the representative’s consent does not mean that the research participant himself/herself is unable to make a judgement. Rather, based on the concept of relational autonomy mentioned earlier, it is important to consider **what kind of support from a representative person will respect the relational autonomy of the research participant**. The **important** points in the selection of a legally acceptable representative can be summarized as follows: (1) Careful judgment should be made as to **whether or not a legally acceptable representative is really necessary** and (2) the **legally acceptable representative should understand the human nature of the research participant and be able to maximize the research participant's benefit.**

What is important here is not whether to obtain consent by the patient or consent by a legally acceptable representative, but rather the creation of a virtuous cycle in which patients with mental or neurological disorders progress toward autonomy with the support of their legally acceptable representatives, while their legally acceptable representatives become better at understanding their intentions through support. This is a virtuous cycle to support the participation of vulnerable research participants. Doctors, nurses, other health care professionals, and medical and other researchers are expected to **mediate the collaborative practice of relational autonomy** between vulnerable research participants, potential participants, and their legally acceptable representatives.

**3-6. Protection of Privacy**

In accordance with the basic spirit of the Act on the Protection of Personal Information, it is necessary in general to protect the personal information of research participants, taking account of their sensitivity. Here, we add a few words about privacy and personal information that are characteristic of brain research. The brain is the seat of the mind, and brain research is about the human mind. The workings of the mind are not easily known by others but can be accessed privately by oneself. Thus, **the workings of the mind** are nothing less than the **privacy of the person himself/herself**. Brain research is an approach to the workings of the mind, which can be kept private. **Researchers involved in brain research must always be aware that their research targets not only the brain but also the mind**. In addition, the protection of personal information in brain research includes, among others, the protection of the shape of the human face, which can be constructed from images obtained by brain imaging research such as MRI on human subjects.

**An incidental finding** is a chance finding that may affect the health of a research participant, such as a malformation of a cerebral blood vessel or a brain tumor discovered by chance in a brain imaging study. The current "Ethical Guidelines for Medical and Health Research Involving Human Subjects" stipulates that the research protocol should clearly state how to respond to such incidental findings when they are discovered. The Strategic Promotion Program for Brain Science Research has proposed the following guideline for the handling of incidental findings. "All brain images taken in the course of brain science research should be screened by physicians to ensure that obvious abnormalities are appropriately detected." Surveys also show that the general public, more so than researchers, would prefer more generous measures for medical support when incidental findings are discovered. In light of this, it is desirable that the research system, to the extent that circumstances permit, pre-determine measures that will **increase the benefits to research participants, such as ensuring access to physicians.**

**4. Special Considerations for Research Participation**

**4-1. Considerations for Research Participants**

If research participants are vulnerable, they will require special considerations and additional safeguards^[[10]](#footnote-10)^ different from those for healthy research participants. It is recommended that such considerations and additional measures be provided not only during the research, but also after its completion.

First, the following considerations and additional safeguards are required **during the research** in terms of **researcher protection and, in particular, risk minimization.**

**Special considerations for vulnerability**: When risks associated with participation in research occur, such as discomfort or itching when wearing a device, discomfort due to side effects of administering a new drug, or other adverse events, vulnerable research participants (e.g., patients with dementia), unlike healthy research participants, may be unable to communicate this to the researchers. The nature of the risk itself may be the same, but the way to deal with it may be different for healthy research participants (by verbal communication) than for vulnerable research participants (by push-button communication).

**Additional safeguards for vulnerability**: In addition to the special considerations described above, additional measures are needed to protect research participants when they are vulnerable, as opposed to when they are healthy. For example, when inpatients with mental or neurological disorders are research participants, it is desirable that physicians make rounds and nurses check their condition more frequently than usual to check for adverse events.

Next, **after the research is completed**, the following considerations are desirable. Although these are also desirable in normal research, in the case of vulnerable research participants, it is desirable to take more thorough measures after the research is completed.

**Ex-post safeguards**: Not all relationships with research participants are terminated upon completion of the research. Research participants are entitled to **appropriate protection and care** after the research is completed. Specifically, it is desirable to establish some sort of **contact point** for research participants to consult with and respond to, if necessary, even after the research has ended.

**Right to know**: Research participants should have access to the results of the research. Therefore, efforts should also be made to **disclose and provide information** on research results **to the greatest extent possible.** Specifically, a notice should be posted on the website when a paper is published.

**4-2. Consideration for Family, Friends, etc.**

As is true for clinical research in general, in the case of vulnerable research participants, consideration of family, friends, and others (family in this context is not limited to legal relatives, but includes **significant others**) is also essential. In the case of mental and neurological disorders, family members and friends of the research participant are often the primary **care providers** for the research participant. In such cases, ethical considerations should include not only the research participants but also their families and friends. As a basic rule, it is advisable to maintain ongoing communication with family members and friends who are the primary caregivers of the participants in the study. This Guide includes special consideration for family and friends as an independent item because it takes seriously the concept of "**relational autonomy**" presented in section 3.

**Substantial guarantee of "right to know"**: In the case of patients with mental and neurological disorders, it is desirable to take into account the possibility that research participants may not be able to access research results on their own, and to actively work to disclose research results and provide information to family members, friends, and other **care providers**. Such efforts will satisfy the **requirement not to exclude family members, friends, etc.,** and will substantially guarantee the "right to know" of research participants.

**Feedback on the research**: You might like to know what concerns (e.g., a sudden change in the person's physical condition) or what satisfaction (e.g., satisfaction with social contribution and altruistic activities) the person had in participating in the research. To know these, you might like to ask not only the research participants but also their families, friends, and other related parties to participate in various ways during and after the research by, e.g., conducting interviews with them. It is also desirable to use this as feedback for future research.

**Other considerations**: If transportation expenses are required for research participation, it is desirable to provide transportation not only for the research participants but also for their care providers. This is because it is often difficult for patients with mental or neurological disorders to come to hospitals (or other research institutions) alone to participate in research, and in such cases, their care providers must accompany them to participate in research. Imposing an additional cost burden on research participants and their family members who are already burdened in the form of participation in research is to place a "double burden" (Ethics Point 2) on them and it is not considered to be ethically appropriate.

**B. A Guide to an Appropriate Ethical Review System**

**5. Appropriate Ethical Review**

Research ethics review has been systematically designed with a focus on medical research involving human subjects. Since 2000, several guidelines for medical research have been established. Currently the "Ethical Guidelines for Medical and Health Research Involving Human Subjects" (established in 2014, revised in 2017) is in operation as a somewhat comprehensive guideline. These guidelines stipulate that researchers must undergo research ethics review before conducting research. The pillars of research ethics review is peer review by fellow researchers and evaluation by an outside party who has no vested interest in the research institution where the ethics review committee is established. Over the past 20 years, the establishment of research ethics review committees at medical universities and hospitals around Japan has expanded rapidly. While there are many brain function studies that can be classified as medical research because they contribute to the future development of medicine, some psychological research and cognitive science research do not need to be classified as medical research. However, even if the research is not clearly classified as medical research, it is advisable to have it reviewed by the research ethics review committee of the institution in accordance with the Ethical Guidelines for Medical and Health Research Involving Human Subjects.

**5-1. The Nature of the Ethics Review Committee**

While it is taken for granted that medical research involving human subjects must be reviewed by an ethics review committee, the following additional considerations are recommended when an ethics review committee reviews research involving patients with mental and neurological disorders. (1) Participation of experts: Participation of experts on the disorder in question. (2) Participation of interested parties: participation of patients with the disorder, their family members, and advocacy groups for their rights. The participation of the above people will enable a more appropriate review of whether reasonable considerations have been made in the research.

Nevertheless, it would be difficult in practice to strictly require the above conditions in the ethics review committees of institutions where research involving patients with mental and neurological disorders is conducted only infrequently. For such institutions, the use of research ethics support as described in the next section (5-2) is recommended.

**5-2. Research Ethics Support for Researchers**

In institutions where it is difficult to obtain the views of experts and stakeholders in mental and neurological disorders within the ethics review committee, it is recommended that individual researchers consult this guide and give consideration to important points at the research protocol stage. In addition, there is a research assistance desk in the ethics section of Strategic Research Program for Brain Sciences (supported by Japan Agency for Medical Research and Development). This service is available if you have any concerns about ethical considerations that arise. Dispelling ethical concerns at the research protocol stage will also help ensure prompt implementation of the research. Please be proactive in using this service.

**5-3. Compliance with the Clinical Trials Act**

The newly enacted "Clinical Trials Act" has been in effect since April 1, 2008. The law requires that research designated as "specified clinical trials" be reviewed by an accredited clinical research review committee and subject to its judgment, along with some additional considerations (e.g., management of conflicts of interest) (i.e., the law does not require anything uniformly of all clinical trials).

Specified clinical trials as defined in this act include "clinical trials on pharmaceuticals not approved or not indicated under the Pharmaceuticals and Medical Devices Law" and "clinical trials on pharmaceuticals funded by pharmaceutical companies, etc.". Research on unapproved drugs or research in collaboration with pharmaceutical companies includes research on mental and neurological disorders. Please undergo ethical review in accordance with the law as necessary.

**Conclusion**

While what we have presented here are short-term measures, we need to improve the social or institutional environment surrounding research on mental and neurological disorders in the long term as well. This Guide makes the following three recommendations in this regard.

First, it is desirable to improve the literacy of researchers and the public regarding research on mental and neurological disorders. We believe it is necessary to continue to develop educational materials for this purpose.

Second, it is desirable to continue attempts to ensure the quality of ethics review committees. To this end, we believe it is necessary to encourage researchers and ethics review committee members to attend various research ethics seminars.

Third, it is desirable to continue to revise this guide to keep pace with the changing environment surrounding research on mental and neurological disorders.

1. Rosenstein and Miller (2008) [↑](#footnote-ref-1)
2. Rothman (1991) [↑](#footnote-ref-2)
3. Chwang (2014) [↑](#footnote-ref-3)
4. Yanos et al. (2009); Taylor et al. (2015) [↑](#footnote-ref-4)
5. DuVal (2004); Nugent et al. (2017) [↑](#footnote-ref-5)
6. Helmchen (2012) [↑](#footnote-ref-6)
7. Binik (2014) [↑](#footnote-ref-7)
8. National Bioethics Advisory Commission (2002) [↑](#footnote-ref-8)
9. Christman and Anderson 2008.; Mackenzie 2008.; Mackenzie and Stoljar 2000. [↑](#footnote-ref-9)
10. Binik and Weijer (2014) [↑](#footnote-ref-10)
